# Hospital Meetings, &c.

**Published:** 1901-02-23

**Authors:** 


### HOSPITAL MEETINGS, &c.

### ROYAL ORTHOPÆDIC HOSPITAL.

The question of rebuilding, on the present site or another, once more occupied a large amount of the attention of the annual meeting of the Royal Orthopædic Hospital, Oxford Street, on Monday last, 18th inst. Mr. H. H. Marks, M.P., the chairman, presided, and moved the adoption of the 62nd report. The ordinary income shown for the year was £1,801, the extraordinary (legacies) £1,610. The ordinary outlay was £2,740, and the extraordinary (furniture, bedding, and linen) £206. Notice had been received of a legacy from the late Mr. J. E. Waddilove, who bequeathed fifty shares in the London Joint-Stock Bank for the purpose of endowing a cot in the hospital in memory of his daughter, Elizabeth Charlotte. This legacy, which will revert to the hospital on the death of the widow, was interesting as being the first endowment received by the charity. The committee expressed satisfaction that the donations showed an increase of £178; but annual subscriptions showed a slight decrease. During the year some of the investments forming the capital funds had been altered, the money being reinvested in approved Colonial securities. Some time since the sanitary condition of the hospital was declared to be unsatisfactory. Under the advice of Professor Corfield and Mr. Keith Young an expenditure of over £2,000 had been made on it, and since the completion of the work no illness from defective drainage had occurred.

Mr. Flower, M.P., the deputy-chairman, seconded the report, and after some discussion with regard to the site, during which the chairman mentioned that the best offer received was of £1,300 a year, with the right to the lessors to buy at 28 years' purchase, it was carried *nem. con.*

The committee of management—with the exception of Mr. Parker, who resigned—was then re-elected, as were the trustees, the treasurer, and one of the hon. auditors, Mr. George Clarke. His colleague, Mr. Rayner, having resigned, received the thanks of the governors, and Mr. A. W. Sulley was elected in his stead. The honorary architect, Mr. Emden, did not come up for re-election, that not being provided for in the rules, but Dr. Walsh raised the question as to whether the appointment was actually, and would continue to be, an honorary one, and after considerable discussion expressed himself satisfied on the point; Mr. Emden declaring that were an honorarium to be at any time voted to him he should at once return it to the hospital. The honorary surgeons were then reappointed; and this would have concluded the business of the ordinary meeting but for a letter which Mr. Reeves, the senior surgeon and a member of the committee, put in, asking that it should be read. This suggested a scheme for obtaining subscriptions by means of a co-operative plan, collectors being given a commission on the sums they obtained, as a memorial to her late Majesty, who granted the charter and was a patron of the charity for 50 years. The chairman permitted the reading of this, but ruled a resolution embodying the scheme out of order, as one of which it would have been necessary to give notice.

The Court was then made special, and a resolution, proposed by the Chairman and seconded by Mr. Flower, empowering the Committee to continue their labours in regard to the discovery of and negotiation for a suitable site, in accordance with the special resolution passed at the Court of Governors on June 12th, 1900.

Mr. Marks said the point at issue between the majority of the committee and Mr. Reeves and his friends was this: that Mr. Reeves thought they should rebuild the hospital on the present site, while the committee thought it should be elsewhere. The present site was worth a rental of £1,500 a year, and if disposed of this should provide them with funds for a new site and for building as well, and should add to their income. Mr. Reeves proposed to rebuild on the present site, and to eke out the income with the rents of shops to be erected on the Oxford Street frontal. No arrangement could, of course, be concluded without the consent of the trustees and of the Charity Commissioners, but the committee desired a remewal of the free hand given them to undertake negotiations.

Mr. Flower, in seconding, reminded the meeting that the Prince of Wales's Fund had given them £250 on condition that they should move to a less expensive site. This year no grant had been made, but a note had been made that the condition of the previous grant had not been fulfilled.

Mr. Reeves argued that the removal of the hospital was not necessary on the ground of non-success in the work; only one death had ever been recorded, and that was certified as from scarlatina, and not from the operation performed.

Dr. Walsh said Mr. Reeves seemed the only consistent advocate of a policy; both the old and the new committees had changed their minds, and eventually disagreed with him. With reference to the advice of the Prince of Wales's Fund, he would remind them that they had also advised the removal of the Moorfields Hospital, and that was now plunged in what looked like financial ruin. He wished to amend the resolution by adding to it that, in the event of its being decided to move, the site be sold at the City Mart, with a month's previous advertisement.

Mr. Martin, the treasurer, said the Mart might be a good place to get the value of property, but not necessarily to sell it. Various objections to the amendment were pointed out, and it failed to find a seconder.

The motion was then put, and declared carried by nine for to four against. The proceedings then terminated, having lasted some hours.

### LONDON FEVER HOSPITAL.

The annual general meeting of governors of the London Fever Hospital was held on the 15th inst. at the Hôtel Victoria, Lord Balfour, president, being in the chair. The report showed that the total number of patients under treatment during the year was 749, exceeding the previous year's total by 49. There were 339 patients admitted with scarlet-fever during the year, and the mortality from this disease was 0·8 per cent., as compared with the general mortality from scarlet-fever in London of 2·6 per cent. There was good evidence that the disease was lessening in severity, for the proportion of fatal cases was decreasing. The hospital was unsupported by rates, and depended for support entirely upon donations and subscriptions and the payments by patients. The funds at command were insufficient to enable the committee to carry out the work of reconstructing the older parts of the hospital, as required by the committee of visitors of the Prince of Wales's Fund. Sir Thomas Barlow, in moving the adoption of the report, urged the claims of the hospital on the public because it upheld the voluntary principle. The motion was carried. The President, in moving that a vote of thanks be tendered to the committee, said that King Edward VII., as Prince of Wales, had been for nearly 40 years patron of the hospital. He moved that a resolution of condolence and sympathy be passed, and forwarded through the proper channel to the King. He hoped that his Majesty would continue as their patron, but they would have to ask for further permission. As regarded the actual work of the hospital, 199 of the patients were treated free of charge. The ordinary fee was paid by 513 patients, and in 15 cases the fees were reduced or remitted altogether. The average number of patients in the hospital was 66, as compared with 75 in the previous year. The demand for the admission of diphtheria cases was increasing, and some applications of this nature had had to be refused. The hospital was suffering from the diversion of annual subscriptions to the Prince of Wales's Fund, which, however, had given them £500 as compared with a grant of £200 last year. He computed that the institution of the fund had resulted in a total loss to them of nearly £2,000. He did not complain. The fund was doing good in the main, but it was prejudicial in their individual case, and crippled their power to reconstruct the older buildings. An expenditure of £15,000 or £20,000 for the improvement of the hospital and for a convalescent home was needed.

### ST. MARY'S HOSPITAL FOR SICK CHILDREN, PLAISTOW.

THE annual Court of Governors was held at St. Mary's Hospital for Sick Children, Plaistow, on Thursday, 14th inst., the Bishop-designate of Barking in the chair.

After the usual preliminaries, the Secretary read the report. The number of patients was shown to be greater than in any previous twelve months, and was as follows:—In-patients 700, out-patients 3,658, casualty patients 13,096. Total number of attendances 59,244. The ophthalmic department, which was first opened in October, 1898, had proved a great boon; the very large number of cases treated, 687—making 6,327 attendances—proved, in the opinion of the Committee, how much this new departure was needed and how highly it was valued by the poor of the district. The hospital, which was originally built for the purpose of a day nursery only, had grown very rapidly. The report stated that the disadvantages of structure would make many alterations necessary, and that if the hospital was to cope successfully with the ever-increasing numbers of those seeking relief it must, before many years had passed, be entirely rebuilt.

To begin with, the out-patients' accommodation, notwithstanding the enlargement of the waiting-room effected in 1899, was insufficient, the crowding of patients in the corridor occasioning many inconveniences; the surgery was cramped and the administrative and domestic offices small and badly situated. These, however, were not the worst evils, the chief difficulty arising with the wards, which, although well ventilated, did not possess as great a cubic air space as was desirable from a hygienic point of view. Special attention had been drawn to the latter fact by the honorary secretaries of the Prince of Wales's Hospital Fund for London, when forwarding the grant for the year. The purchase of the mission hall adjoining, and the building of a new hospital, would entail the expenditure of probably £12,000 or thereabouts, and towards this amount £768 11s. 11d. only had been subscribed. The new mortuary referred to in the annual report for 1899 had been duly erected and equipped, the cost being £205 2s. 7d. The Committee, after mature consideration, felt that, owing to the cottages which were used as a laundry not being the property of the institution, and to the fact that any structural alterations would, of necessity, involve their purchase, it would be advisable to place out the whole of the washing: this had accordingly been done, and it was hoped that the annual cost would not, in consequence, be greater than hitherto.

Financially the past year had been on the whole satisfactory, the general fund closing with a balance of £51 2s. 5d. in hand, after meeting all liabilities and repaying the building fund £158 11s. 2d., which was the final instalment of the amount borrowed in 1898. The assured income of the hospital, however, amounted only to £47 (derived from investments on account of two cot endowments), against an average expenditure of £3,500, and an earnest appeal was made to subscribers and friends to enable the hospital to steer clear of debt.

The sad death of Dr. E. A. T. Steele, which took place in July last, deprived the hospital of a sincere friend, who had done much in its interests and contributed largely to its efficiency. He had been connected with the institution from its foundation.

Dr. J. W. H. Eyre, who had so ably filled the office of honorary ophthalmic surgeon, felt compelled to resign owing to the pressure of work entailed by other professional appointments, but had consented to remain on the staff as honorary bacteriologist; he had been succeeded by Mr. Kenneth Scott. Dr. Cuthbert Lockyer had been elected to the vacancy caused by the death of Dr. Steele, and would devote his attention to gynæcological work.

The following changes had taken place on the resident staff:—Dr. T. B. Sellors succeeded Dr. J. C. S. Peatson as resident medical officer; and Dr. J. Alcindor, and subsequently Dr. R. Sidney Ellis, succeeded Dr. R. M. Ralph as assistant resident medical officer. Mr. Percy Glenton had been elected secretary, and Miss T. A. Bodington matron. By the retirement of Miss L. K. Given-Wilson, who was secretary since the commencement of the hospital, and by that of Miss E. E. Simmonds, matron since 1896, the committee lost the assistance of two exceptionally valuable officers. On the motion of Mr. Alexander, seconded by the Rev. T. Given-Wilson, the report was adopted unanimously. The audited accounts were also adopted unanimously, on the motion of Dr. Randall, seconded by the Rev. T. Given-Wilson. Representatives were then elected for the Committee of Management for 1901 as follows:—Captain Matthews, Mr. J. Templey, Dr. Randall, Canon Pelly, Mr. David Howard, J.P., and Mr. J. Weaver Smith. The Governors present were:—Canon Pelley (Vicar of West Ham), Mr. Hoppe, Mr. Alexander, Mr. W. Neave Hill, and the Rev. T. Given-Wilson.

### CITY OF LONDON LYING-IN HOSPITAL.

THE annual Court of Governors of the City of London Lying-in Hospital was held on Wednesday, 13th inst., Mr. Andrew R. Motion, J.P., in the chair. The 150th annual report, which was taken as read, showed that the past year had been a satisfactory one, both as regarded the sanitary condition of the hospital and its financial position. There had been admitted to the benefits of the hospital:—As in-patients, 599; as out-patients, 1,753; a total of 2,352, being the largest number of patients the committee had ever had to report as attended in one year.

The 599 women (being 14 more than last year) delivered in the hospital gave birth to 611 children, viz.: 308 boys and 303 girls; 10 women had twins, and one woman triplets; 31 children were stillborn, 21 of these being either decomposed or premature, 16 apparently stillborn but restored, and 20 infants died, 12 of these deaths being due to premature birth.

The one death that took place among the women admitted the Court felt should hardly be counted as a hospital case at all; it was that of a woman sent from Edmonton by a local practitioner. On arrival at the hospital the patient was in such a critical condition that admission could not be refused. Labour was artificially induced, and she was delivered of a stillborn child which had been dead several days; the mother died from exhaustion 36 hours after delivery.

The daily average number of beds occupied was 26·5, and the average stay of each patient 16 days; the average daily number of inmates was 72·8, as against 69·4 in 1899. On the recommendation of the "Soldiers' and Sailors' Families' Association" 19 in-patients and 29 out-patients were treated without a subscriber's letter. 1,753 women were delivered by the midwives at their own homes; they were from the districts of Shoreditch and Hoxton, Islington and Holloway, Bethnal Green, St. Luke's, Clerkenwell, Stoke Newington and South Tottenham, Hackney, City, Spitalfields and Whitechapel. Of these cases 2 women and 31 children died. The midwife pupils attended 745 of the above, with the district surgeons and resident outdoor midwife. Forty-four midwives and 168 monthly nurses were trained during the year; 41 of the midwives presented themselves for the London Obstetrical Society's Examination and passed with two exceptions. The total cost of the new building containing the new dormitories and sitting-rooms for nurses, including furnishing, amounted to £3,460, and in moving the adoption of the report and balance-sheet the Chairman said it was satisfactory that it had only been necessary to draw upon the capital account for £1,000. In spite of many calls, war, &c., the income for 1900 amounted to £4,705 17s. 11d.

The Rev. S. Buss seconded; he thought that the hospital was in a very satisfactory condition—they were fortunate in their matron; the number of deaths compared most favourably with that of past years, and with the record of other hospitals. The report and balance-sheet were adopted unanimously.

The Rev. G. H. Perry moved the adoption of the medical report, which stated, in addition to the particulars given in the general report, that premature labour had been induced 9 times—in 5 cases the child was alive, and in 4 stillborn. It was the custom for women of the class treated to call in their own medical man at the last extremity; he, finding the case a difficult one, put the patient in a cab and sent her to the hospital. During the year three women had been delivered in cabs, one in a tramcar, and one in the street on the way to the hospital. The causes of death were: in the one case mentioned as occurring in the hospital, puerperal eclampsia; and in those of the two out-patients, one was from acute bronchitis, and the other from puerperal septicæmia.

Mr. J. J. Paterson seconded, and the medical report was adopted unanimously.

Mr. W. P. Russell moved the re-election of the committee of management, with the addition of the Rev. J. Selden, and that it be left to the committee to fill up any vacancies occurring during the year. He had always advocated the extension of the radius for out-patients, and commended this point to the consideration of the committee. Mr. Collins seconded the proposal, and the committee was re-elected. The finance committee and hon. officers having been re-elected, Mr. Pedder proposed the re-election of Dr. Yarrow as Surgeon-Accoucheur. He had known Dr. Yarrow, who had just ceased to be the Medical Officer for the Parish of St. Luke, for thirty years. Mr. Collins seconded, and Dr. Yarrow was declared re-elected.

Dr. Yarrow replied. He could not promise to do better than in the past; he thought such an unfortunate case as the one mentioned in the report must be regarded as an accident, and looked upon as an accident case would be in a general hospital. Such cases had no claim on the hospital, but their condition was so extreme that they could not be sent away. He drew attention to the fact that of four such cases only one had died.

The usual vote of thanks to the chairman ended the proceedings.

### HOSPITAL FOR DISEASES OF THE THROAT.

The annual general meeting of the Hospital for Diseases of the Throat, Golden Square, was held at the Medical Society's Rooms on Wednesday, 13th inst., Mr. G. H. Pember presiding. A resolution was passed expressing the sorrow of the meeting at the death of her late Majesty, and congratulating the King on his accession to the Throne. The report of the Committee of Management and Accounts for the past year were then submitted. From these it appeared that the ordinary receipts were £5,954, and the ordinary expenditure £5,233. There was, however, a loan from the bankers of £2,500, and of this £1,500 had been repaid, while £2,805 was expended on building improvements. The subscriptions for the year were £298, as against £325 for 1899, and the donations were £2,029 as compared with £4,611—the latter sum, however, included £3,823 from the Special Dinner Fund Appeal. During the year 10,383 new out-patients were treated, the total attendance being 42,866. The in-patients numbered 569. The £1,500 of the loan from the bankers was repaid as a result of a special appeal made by the president, Lord Rothschild, and the hope was expressed that the friends of the hospital would do their best to clear off the remainder. The committee reported with regret, that Miss Bywater, who had been matron since 1894, had resigned her appointment, the vacancy being filled by the selection of Miss Marwood of Charing Cross Hospital. The report was adopted. Of the six retiring members of the committee, two, Mr. Jackson and Colonel Clifford Probyn, were re-elected. To fill the vacancies Messrs. John Andrew Coland, Percy Browning Edwards, and George Ingram were elected. The treasurer, Mr. C. J. Lacy, was also re-elected with acclamation and observed, in returning thanks for the compliment, that he had been a member of the committee for 34 years, and treasurer the greater part of that time. They were, he regretted to say, very short of cash. An institution like theirs ought not to be in the position of having to pass large bills on to the next committee meeting for lack of funds to meet it.

The auditors, Messrs. Ford, Rhodes and Ford, having been re-elected, the chairman moved a resolution to alter By-law 21 by adding the words "two senior members of the active medical staff," the effect of which would be to admit of the two senior members of the hon. medical staff being *ex officio* members of the Committee of Management, in addition to the Dean of the Medical Council, who is already an *ex officio* member. The resolution, he said, had received the unanimous approval of the committee and had not been brought forward without long and earnest consideration. However able the Dean might be it was hardly possible for him always to represent, or even gauge the feeling of the whole staff. The resolution was carried *nem. con.*, and votes of thanks brought the proceedings to a close.

### ST. MARK'S HOSPITAL.

In the absence of the Lord Mayor Mr. Richard Biddulph Martin, the treasurer, took the chair at the sixty-fifth annual meeting of St. Mark's Hospital at the Mansion House on Thursday, 14th instant. From the report and accounts presented it appeared that there were 430 in-patients admitted during the year, of whom 353 were discharged cured, 54 relieved, and 3 unsuitable. Operations were refused in 5 cases, 4 were transferred to other hospitals, 4 died, while 28 remained under treatment at the end of the year. There were 1,171 out-patients, with 5,212 attendances; while 100 cases were waiting for admission as in-patients. The ordinary income was £2,123 and the extraordinary income (legacies) £1,789. The ordinary expenditure was £4,075, and the extraordinary expenditure (repairs, new boiler, &c.) £425. The death was reported with regret of Mr. Geo. A. Whealler, for upwards of 30 years a member of the committee, and of Sir A. Bevan and Mr. E. Allcard. Mr. F. W. Haigh's resignation as a member of the committee and the election of Mr. Arthur Levita were also mentioned. Thanks were tendered to the Prince of Wales's Fund and the Saturday Fund for grants of £150 and £146 respectively, but regret was expressed that the Drapers' Company had withdrawn the support they had accorded for over 30 years in favour of the Prince's Fund. In consideration of grants amounting to upwards of £1,000 the committee had dedicated a bed to the Grocers' Company in the "Holgate Foster" ward. The report was adopted, and after the election of Mr. J. Lea Smith to the committee and the re-election of the retiring members, votes of thanks brought the proceedings to a close.

